# Emerging challenges in evidence-based public health and how to address them

**DOI:** 10.25646/6500

**Published:** 2020-06-04

**Authors:** Mark Petticrew

**Affiliations:** London School of Hygiene and Tropical Medicine, Department of Social and Environmental Health Research

Public health has been defined as ‘the process of mobilizing and engaging local, state, national, and international resources to assure the conditions in which people can be healthy’ [1].

‘Evidence-based public health’ aims to put scientific evidence at the core of this process, integrating it into public health decision-making, alongside other evidence and expertise [2]. This is of course challenging. One reason is that public health evidence derives from many sources, collected using a wide range of scientific methods. That evidence is often difficult to produce, and generalise from. This can make primary research (e.g. evaluations), and secondary research (e.g. systematic reviews), difficult and costly. Straightforward epidemiological concepts – even apparently simple ones like ‘population,’ ‘intervention’ and ‘outcome’ – can also be more difficult to define in public health contexts [3].

Overall, then, this has been one of the greatest challenges facing public health: how to develop and use meaningful actionable evidence. In the face of such complexity, researchers often retreat into using simpler approaches. However simplistic models of evidence and evaluation are often misleading, and can even be harmful. In contrast, conceptualising interventions as changes in complex systems (rather than as discrete, bounded events) can help with the development and implementation of public health interventions. Systems-based approaches require that we think about public health evidence and public health methods quite differently. This can be a challenge in itself – but such systems-based approaches offer great promise [4]. Not least, they can help us to think about how to implement evidence across very different contexts.

This is of practical importance because many non-communicable diseases (NCDs, and their related inequalities) arise from systems. Such systems include markets for unhealthy commodities – such as tobacco, alcohol, unhealthy food, gambling and many others. For example it has been argued that if we want to understand why people are consuming more unhealthy foods – a major public health challenge in low and middle income countries, as well as in better-off countries – we need to study the transformations to economic and social systems that are favouring their increasing availability and affordability [5].

This presentation will aim to describe how applying a complex systems lens to public health can help address major public health problems (like NCDs), but can also help us create more useful, actionable evidence. It will use examples from tobacco, alcohol, and food, among others [6, 7].
